# Efficacy and Safety of Prolonged Dual Antiplatelet Therapy after Percutaneous Coronary Intervention in Acute Coronary Syndrome Patients

**DOI:** 10.5334/gh.1185

**Published:** 2023-03-13

**Authors:** Jialun Han, Yi Zhang, Xiujin Shi, Baidi Lin, Yunnan Zhang, Ru Zhang, Yifan Wang, Jialin Yan, Yang Lin

**Affiliations:** 1 Department of Pharmacy, Beijing Anzhen Hospital, Beijing, China; 2 School of Pharmaceutical Sciences, Capital Medical University, Beijing, China; 3 Department of Pharmacy, Beijing Chaoyang Hospital, Capital Medical University, Beijing, China; 4 Department of Pharmacy, Wenzhou Central Hospital, Wenzhou, China; 5 Department of Pharmacy, Shougang Hospital, Health Science Centre, Peking University, Beijing, China

**Keywords:** acute coronary syndromes, dual antiplatelet therapy, percutaneous coronary intervention, ticagrelor

## Abstract

**Objective:**

It remains controversial whether to extend the course of dual antiplatelet therapy (DAPT) after percutaneous coronary intervention (PCI). We conducted a study to investigate the benefits and risks of applying DAPT for different durations after PCI in acute coronary syndromes (ACS) patients in China. What’s more, we explored the efficacy of extended DAPT regimen based on ticagrelor.

**Methods:**

This single-center prospective cohort study used data obtained from the PHARM-ACS Patient Registration Database. We included all patients who were discharged between April and December 2018. All patients had at least 18 months of follow-up. Patients were divided into two groups according to the duration of DAPT: a 1-year group and a >1-year group. Potential bias between the two groups was adjusted for by propensity score matching using logistic regression. The primary outcomes were major adverse cardiovascular and cerebrovascular events (MACCE), defined as a composite of death, myocardial infarction, and stroke occurring from 12 months after discharge to follow-up visit. The safety endpoint was any significant bleeding event (BARC ≥ 2).

**Results:**

Of 3,205 patients enrolled, 2,201 (68.67%) had DAPT prolonged beyond one year. A total of 2,000 patients were successfully propensity score-matched; patients who received DAPT > 1-year (n = 1000), compared with DAPT = 1-year patients (n = 1000), had a similar risk of MACCE (adjusted HR 0.23, 95% CI 0.05–1.10) and significant bleeding events (adjusted HR 0.63, 95% CI 0.32–1.24). The DAPT > 1-year group had a higher risk of revascularization (adjusted HR 3.36, 95% CI 1.64–6.87).

**Conclusion:**

Prolonged DAPT may not be of sufficient benefit to ACS patients within 12–18 months after the index PCI to offset the increased risk of significant bleeding events.

## Introduction

Acute coronary syndromes (ACS) are one of the leading causes of death in patients with coronary heart disease. Percutaneous coronary intervention (PCI) is the most important method to treat coronary heart disease, especially ACS. According to the ESC and ACC/AHA guidelines, dual antiplatelet therapy (DAPT) is a vital medication regimen for ACS patients after PCI [1,2,3]. Nevertheless, for DAPT beyond 12 months after PCI, the guideline recommendation level and level of evidence remain inadequate. Chinese guidelines and expert consensus make similar recommendations [4,5].

Debate remains on whether to continue DAPT for more than one year [6]. A meta-analysis incorporating randomized controlled studies argued that a longer course of DAPT is related to a lower risk of myocardial infarction (MI) and increased risk of major bleeding events [7]. Nevertheless, recent research based on real-world data puts a different spin on this view [8,9,10]. They found that extended DAPT course failed to provide benefit for patients.

The novel antiplatelet agent ticagrelor has a more obvious platelet inhibitory effect than does clopidogrel. The newest guidelines recommend ticagrelor as a first-line agent for secondary prevention of ischemic events in patients with ACS [1,2,3]. Previous studies have rarely involved ticagrelor, except for the PEGASUS-TIMI54 study, which showed that ticagrelor combined with aspirin significantly reduced the risk of cardiovascular death, MI, or stroke in patients 1–3 years after MI [11]. Moreover, because of the widespread clinical use of clopidogrel, there is insufficient real-world evidence to establish the risks and benefits of long-term use of DAPT based on ticagrelor.

Recent studies have found that prolonged DAPT is more common than the 1-year regimen in Chinese ACS patients [12], and the effectiveness and safety of this treatment pattern require urgent verification. This study aims to investigate the benefits and risks of applying different courses of DAPT after PCI in ACS patients through a real-world observational study. At the same time, we explored prolonged DAPT with ticagrelor in the real-world and provides new evidence for making clinical decisions in these patients.

## Methods

### Study design and population

This study is a retrospective analysis of data from the PHARM-ACS registry (NCT04184583). PHARM-ACS is an ambispective (retrospective and prospective) single-center ongoing observational registry study conducted by the Department of Pharmacy, Beijing Anzhen Hospital on pharmacotherapy and long-term clinical outcomes in patients with ACS after PCI. This registry includes adult patients (≥18 years old) who were discharged from Beijing Anzhen Hospital between April 2018 and December 2021. All patients were diagnosed with ST-segment elevation MI (STEMI), non-ST-segment elevation MI (NSTEMI), or unstable angina and underwent successful index PCI. The ethics committee of Beijing Anzhen Hospital approved the research protocol. All registered patients signed informed consent forms. Our previous studies have described the database in detail [13,14].

We integrated the data of patients from the PHARM-ACS registry if they met the following inclusion criteria: 1) have received DAPT for at least one year; 2) have been followed up for at least 18 months; 3) received clopidogrel or ticagrelor plus aspirin at discharge; and 4) were discharged before December 2018. Patients with the following primary exclusion criteria were excluded: 1) major adverse cardiovascular and cerebrovascular events (MACCE), target vessel revascularization, stent thrombosis, or major bleeding event (Bleeding Academic Research Consortium criteria (BARC) ≥ 2) within 12 months after PCI; 2) treatment with three or more antiplatelet agents; 3) treatment with anticoagulants; or 4) alteration of antiplatelet drugs after discharge. Because prasugrel is not a listed medication in China, all of our patients used clopidogrel or ticagrelor combined with aspirin for DAPT. The enrolled patients were divided into two groups: DAPT = 1-year group and DAPT > 1-year group, according to their duration of DAPT.

### Clinical outcomes

The primary endpoint was the composite of MACCE (defined as all-cause death, MI, and stroke) from 12 months after PCI to the follow-up visit. The secondary endpoints were all-cause death, cardiogenic death, MI, stroke, target vessel revascularization, and stent thrombosis. The safety endpoint was significant bleeding events (defined as bleeding events of BARC ≥ 2).

### Follow-up

Follow-up visits were conducted by well-trained staff every six months. Follow-up channels included telephone, WeChat, and clinical visits. A standard case report form was used to document information on clinical outcomes, medication use, and other drug-related adverse events. The investigator determined endpoint events with standard definitions at each visit. For each uncertain endpoint event, a panel of experts made a judgment based on detailed documentation of the visit.

### Statistical analysis

For inter-group differences, the chi-square test or Fisher’s exact test was used for categorical variables, and the independent sample T-test was conducted for continuous variables. Continuous variables were expressed as mean ± standard deviation and categorical variables as frequencies. Propensity score matching (PSM) was used to adjust for the inter-group differences in baseline characteristics and potential confounding factors. The probability of two groups (propensity score) was estimated by logistic regression. The assessed variables included sex, age, BMI, prior PCI, prior bypass graft (CABG), prior MI, prior stroke, hypertension, diabetes, hyperlipidemia, family history of coronary artery disease, smoking status, ACS diagnosis, number of stents, left main disease, and the type of P2Y12 inhibitor used. The cumulative incidence of clinical events after PSM was assessed by Kaplan-Meier analysis and log-rank test. The multivariate Cox proportional hazards model risk ratio (HR) and 95% confidence interval (CI) were calculated, with variables including age, sex, body mass index, diagnosis of ACS, hypertension, hyperlipidemia, diabetes, current smoker, prior MI, prior PCI, prior CABG, prior stroke, number of stents, number of lesions, and left main disease. The result of the multivariate Cox regression model in the integral study population was shown as unadjusted HR, and in the PSM population was displayed as adjusted HR. The same model was used to estimate the p values for interactions in the subgroup analysis.

All reported p values were two-sided, and p < 0.05 indicated statistical significance for all analyses. Statistical analysis was performed using R version 4.1.0.

## Results

The PHARM-ACS registry included 5,387 patients at the time of data processing. We excluded 1,977 patients with less than 18 months of follow-up; 173 patients had MACCE, revascularization, stent thrombosis, or BARC ≥ 2 bleeding events within 12 months; 32 patients used DAPT for less than one year. This study included a total of 3,205 patients. From PCI to the last follow-up visit, the median follow-up time and interquartile range (IQR) were 572 (IQR 545, 622) days. Among them, 1,004 (31.33%) patients took DAPT for 1 year; 2,201 (68.67%) received DAPT for more than one year, the median DAPT time was 19 (IQR 18, 20) months with 1,886 (58.85%) patients were continuing DAPT at our last follow-up. There were 1,236 (38.56%) DAPT patients who used ticagrelor combined with aspirin, and the remaining patients used clopidogrel combined with aspirin.

The baseline and procedural characteristics of the patients during their hospitalization are shown in Table 1. The baseline characteristics of patients in the two groups are similar (P > 0.05), except that patients in the DAPT > 1-year group had a higher proportion of prior PCI (15.24% vs. 18.90%, P = 0.012), prior CABG (0.50% vs. 1.59%, P = 0.010), prior MI (5.68% vs. 8.54%, P = 0.005) and diabetes (28.19% vs. 33.35%, P = 0.004). The number of stents placed in each patient differed between the two groups (1.56 ± 1.00 vs. 1.65 ± 1.11, respectively, P = 0.039).

**Table 1:** Baseline patient characteristics of the overall cohort and the propensity-score-matched cohort.


	THE TOTAL STUDY COHORT			THE PROPENSITY-SCORE-MATCHED COHORT		
	
	DAPT > 1-YEAR(N = 2201)	DAPT = 1-YEAR(N = 1004)	P VALUE	DAPT > 1-YEAR(N = 1000)	DAPT = 1-YEAR(N = 1000)	P VALUE

Demographics						

Age (y)	60.27 ± 10.06	58.67 ± 10.24	<0.001	58.83 ± 10.26	58.75 ± 10.17	0.862

Male	1658 (75.33)	759 (75.60)	0.870	766 (76.60)	755 (75.50)	0.564

BMI (kg/m^2^)	25.94 ± 3.20	25.71 ± 3.00	0.063	25.91 ± 3.28	25.70 ± 3.00	0.157

Medical history						

Prior PCI	807 (36.67)	328 (32.67)	0.028	318 (31.80)	327 (32.70)	0.667

Prior CABG	63 (2.86)	13 (1.29)	0.007	22 (2.20)	13 (1.30)	0.125

Prior MI	188 (8.54)	57 (5.68)	0.005	64 (6.40)	57 (5.70)	0.511

Prior Stroke	183 (8.31)	75 (7.47)	0.415	72 (7.20)	75 (7.50)	0.797

Risk factors						

Hypertension	1345 (61.11)	604 (60.16)	0.610	593(59.30)	603 (60.30)	0.648

Diabetes	734 (33.35)	283 (28.19)	0.004	300 (30.00)	283 (28.30)	0.403

Hyperlipidemia	809 (36.76)	358 (35.66)	0.549	352 (35.20)	357 (35.70)	0.815

Family history of CAD	99 (4.50)	38 (3.78)	0.355	30 (3.00)	38 (3.80)	0.324

Current smoker	723 (32.85)	320 (31.87)	0.584	329 (32.90)	320 (32.00)	0.667

Examination						

LVEF (%)	61.96 ± 7.80	62.27 ± 7.60	0.359	62.18 ± 7.84	62.26 ± 7.60	0.830

WBC (*10^9^/L)	7.07 ± 1.98	7.07 ± 2.06	0.985	7.21 ± 2.14	7.07 ± 2.06	0.136

Hemoglobin (g/L)	140.84 ± 14.90	140.93 ± 14.69	0.879	141.37 ± 14.26	140.89 ± 14.70	0.467

Crcl (mL/min)	100.13 ± 30.811	101.95 ± 29.68	0.136	102.40 ± 31.45	101.80 ± 29.65	0.673

Clinical presentation						

STEMI	326 (14.81)	142 (14.14)	0.063	131 (13.10)	142 (14.20)	0.194

NSTEMI	362 (16.45)	135 (13.45)		163 (16.30)	135 (13.50)	

Unstable angina	1513 (68.74)	727 (72.41)		706 (70.60)	723 (72.30)	

PTCA only	184 (8.36)	69 (6.87)	0.148	52 (5.20)	69 (6.90)	0.111

Diffuse lesion	665 (30.21)	309 (30.78)	0.748	291 (29.10)	308 (30.80)	0.407

Occlusion disease	574 (26.08)	250 (24.90)	0.479	227 (22.70)	248 (24.80)	0.270

Bifurcation disease	432 (19.63)	205 (20.42)	0.603	183 (18.30)	204 (20.40)	0.235

Diseased vessels						

Left main coronary artery	197 (8.95)	74 (7.37)	0.136	82 (8.20)	74 (7.40)	0.505

Left anterior descending artery	1706 (77.51)	775 (77.19)	0.841	786 (78.60)	772 (77.20)	0.451

Left circumflex artery	1275 (57.93)	553 (55.08)	0.131	562 (56.20)	551 (55.10)	0.621

Right coronary artery	1192 (54.16)	517 (51.49)	0.161	497 (49.70)	515 (51.50)	0.421

Number of diseased vessels						

1	749 (34.03)	374 (37.25)	0.107	359 (35.90)	373 (37.30)	0.596

2	806 (36.62)	367 (36.55)		387 (38.70)	365 (36.50)	

3	646 (29.35)	263 (26.20)		254 (25.40)	262 (26.20)	

No. of stents per patient	1.65 ± 1.11	1.56 ± 1.00	0.039	1.53 ± 0.96	1.57 ± 1.00	0.337

Ticagralor	835 (37.94)	401 (39.94)	0.280	381 (38.10)	397 (39.70)	0.463

β-blocker	1568 (71.24)	685 (68.23)	0.083	720 (72.00)	682 (68.20)	0.063

ACEI/ARB	913 (41.48)	383 (38.15)	0.074	410 (41.00)	383 (38.30)	0.217

Statins	2153 (97.82%)	980 (97.61%)	0.710	977 (97.70)	978 (97.80)	0.880


Values are n (%) or mean ± SD. BMI, body mass index; PCI, percutaneous coronary intervention; CABG, coronary artery bypass graft; MI, myocardial infarction; CAD, coronary artery disease; DAPT, dual antiplatelet therapy; LVEF, left ventricular ejection fraction; WBC, White blood cell count; STEMI, ST segment–elevation myocardial infarction; NSTEMI, non-ST segment–elevation myocardial infarction; PTCA, Percutaneous transluminal coronary angioplasty; ACEI, angiotensin-converting enzyme inhibitor; ARB, angiotensin receptor blocker; PCI, percutaneous coronary intervention.

After 1:1 PSM, 1000 well-matched pairs of patients were obtained, with no significant differences (P > 0.05) in baseline and procedural characteristics between the two groups.

During the follow-up period from 12 months after PCI to 18 months after PCI, a total of 31 (0.97%) patients had MACCE, and 59 (1.84%) patients had BARC ≥ 2 bleeding events. After PSM, 13 (1.30%) cases of MACCE and 24 (2.40%) cases of significant bleeding events occurred in the DAPT = 1-year group, and 5 (0.50%) cases of MACCE and 16 (1.60%) cases of significant bleeding events occurred in the DAPT > 1-year group. There were no significant differences between the DAPT = 1-year and DAPT > 1-year groups in the unadjusted incidence of MACCE (unadjusted HR 0.58, 95% CI 0.27–1.23; adjusted HR 0.23, 95% CI 0.05–1.10) or significant bleeding events (unadjusted HR 1.58, 95% CI 0.99–2.54; adjusted HR 0.63, 95% CI 0.32–1.24) when analyzed by multivariate Cox risk model, as shown in Table 2. While there was no significant difference in the risk of all-cause death, cardiac death, MI, stroke, or stent thrombosis between the two groups, the DAPT > 1-year group had a higher risk of revascularization (unadjusted HR 2.97, 95% CI 1.52–5.78; adjusted HR 3.36, 95% CI 1.64–6.87).

**Table 2:** Clinical outcomes at 1-year before and after propensity score matching.


	THE TOTAL STUDY COHORT				THE PROPENSITY-SCORE-MATCHED COHORT			

	DAPT > 1Y (N = 2201)	DAPT = 1Y (N = 1004)	UNADJUSTED HR (95% CI)	P VALUE	DAPT > 1Y (N = 1000)	DAPT = 1Y (N = 1000)	ADJUSTED HR (95% CI)	P VALUE

MACCE	18 (0.82)	13 (1.29)	0.58 (0.27–1.23)	0.152	5 (0.50)	13 (1.30)	0.23 (0.05–1.10)	0.065

All cause death	7 (0.32)	7 (0.70)	0.35 (0.11–1.17)	0.089	2 (0.20)	7 (0.70)	0.14 (0.02–1.20)	0.073

Cardiac death	4 (0.18)	2 (0.20)	0.51 (0.07–3.80)	0.513	2 (0.20)	2 (0.20)	0.39 (0.03–4.80)	0.465

MI	2 (0.09)	1 (0.10)	0.95 (0.09–10.61)	0.969	1 (0.10)	1 (0.10)	1.00 (0.06–16.02)	0.999

Stroke	9 (0.41)	5 (0.50)	0.81 (0.27–2.47)	0.717	2 (0.20)	5 (0.50)	0.38 (0.07–2.04)	0.262

Revascularization	70 (3.18)	10 (1.00)	2.97 (1.52–5.78)	0.001	32 (3.20)	10 (1.00)	3.36 (1.64–6.87)	0.001

Stent thrombosis	16 (0.72)	4 (0.39)	1.80 (0.60–5.42)	0.294	7 (0.69)	4 (0.40)	1.88 (0.54–6.51)	0.317

Bleeding BARC ≥ 2	35 (1.59)	24 (2.39)	0.70 (0.40–1.20)	0.195	16 (1.60)	24 (2.40)	0.63 (0.32–1.24)	0.179


Values are n (%). CI, confidence interval; DAPT, dual antiplatelet therapy; HR, hazard ratio; MACCE, major adverse cardiovascular and cerebrovascular events; MI, myocardial infarction.

**Figure 1:**
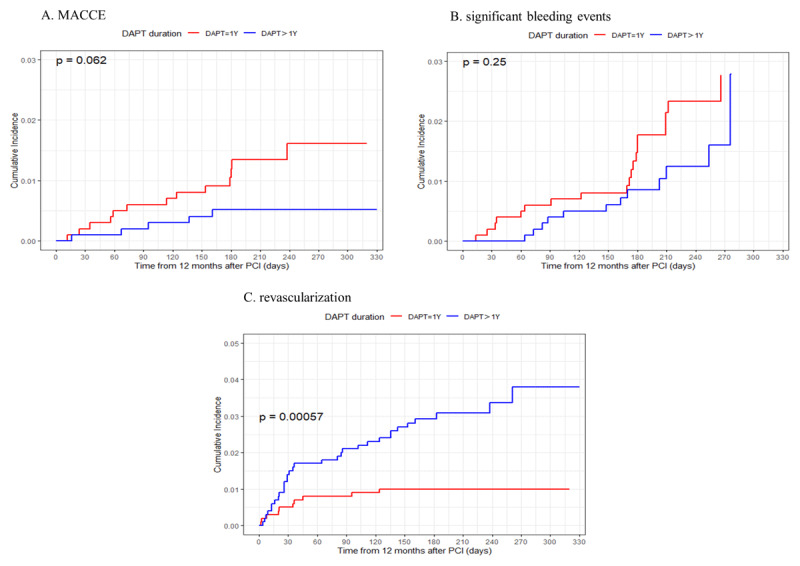
Kaplan-Meier Estimate of outcome events according to DAPT duration. **A:** Propensity-score-matched cumulative incidence of MACCE during the period from 12 to 18 months according to study group. **B:** Propensity-score-matched cumulative incidence of significant bleeding events C: Propensity-score-matched cumulative incidence of revascularization; MACCE, major adverse cardiovascular and cerebrovascular events.

Survival analysis of the Kaplan-Meier curve revealed differences in the cumulative incidence of MACCE (log-rank P = 0.062, power = 0.71), significant bleeding events (log-rank P = 0.25), and revascularization (log-rank P < 0.001) between the two groups, as shown in Figure 1.

The subgroup analysis of MACCE risk data after PSM showed no significant difference in treatment effect between DAPT = 1-year and DAPT > 1-year, even in patients using different P2Y12 receptor inhibitors (P-value for interaction 0.468), as shown in Table 3.

**Table 3:** Subgroup analysis for major adverse cardiovascular and cerebrovascular events during the period from 12 to 24 months in the propensity-score-matched sample.


	DAPT > 1Y (N = 1000)	DAPT = 1Y (N = 1000)	ADJUSTED HR (95% CI)	INTERACTIVE P VALUE

P2Y12 receptor inhibitor				0.468

Clopidogrel	3/619 (0.48)	10/603 (1.66)	0.30 (0.08–1.08)	

Ticagrelor	2/381 (0.52)	3/397 (0.76)	0.56 (0.10–3.76)	

Age				0.703

>60	3/470 (0.64)	9/456 (1.97)	0.32 (0.09–1.19)	

≤60	2/530 (0.38)	4/544 (0.74)	0.47 (0.09–2.59)	

Diabetes				0.833

Yes	1/300 (0.33)	3/283 (1.06)	0.41 (0.13–1.31)	

No	4/700 (0.57)	10/717 (1.39)	0.31 (0.03–3.00)	

Smoke				0.165

Yes	1/329 (0.30)	7/320 (2.19)	0.14 (0.02–1.11)	

No	4/671 (0.60)	6/680 (0.88)	0.71 (0.20–2.53)	

Renal dysfunction				0.904

Yes	2/219 (0.91)	5/220 (2.27)	0.40 (0.08–2.07)	

No	3/781 (0.38)	8/780 (1.03)	0.36 (0.10–1.37)	


## Discussion

This study investigated whether prolonged DAPT for more than 12 months after PCI could provide benefits to ACS patients. We found that compared with 12 months DAPT, prolonged DAPT treatment was not associated with a reduced risk of MACCE or an increased risk of bleeding events with BARC ≥ 2 within 18 months after PCI in patients with ACS. Our study is the first real-world study to explore prolonged DAPT using ticagrelor in a Chinese population, and provides new evidence for making clinical decisions in these patients.

Use of DAPT for more than 12 months is more prevalent than the standard regimen in China (12), as reflected in our study (68.67% patients received DAPT for more than one year). We raised a question about the rationality of this general practice, considering that prolonged DAPT has not been shown to provide sufficient clinical benefit to ACS patients after PCI. Current guideline recommendations for DAPT beyond 12 months remain ambiguous, although ACS patients are still exposed to risk 12 months after PCI [1,2,3,4]. In a high-quality meta-analysis, researchers defined long-term DAPT as ≥18 months [15]. They found that ≥18 months of DAPT resulted in higher rates of non-cardiac death. They excluded the SMART-DATE trial which reported long term arm as 12–18 months DAPT from their sensitivity analysis. To some extent, they ignored the prolonged DAPT regimen within 12–18 months. On the basis of opinion from the previous study, we focused on prolonged DAPT treatment within 18 months in this study. Our study suggests that prolonging the duration of DAPT may not provide sufficient clinical benefit for most patients. At the same time, the increased incidence of target revascularization and long-term medication use may impose a heavier economic burden on ACS patients.

Different views have been drawn from several randomized controlled studies. The well-known DAPT study [16] argued that extending DAPT time can significantly reduce the incidence of MACCE with a higher incidence of moderate and severe bleeding on the GUSTO scale, but no statistically significant difference in severe and fatal bleeding. Furthermore, some studies have highlighted the reduced risk of ischemia and the increased risk of bleeding associated with prolonged DAPT. Khan et al. systematically evaluated several randomized controlled studies and found that extended-term DAPT was associated with a reduced risk of MI (RR 0.68, 95% CI 0.54–0.87) [7]. Yin et al. provided a similar finding [15]. Nevertheless, both the DES-LATE study [17] (2.4% vs. 2.6%) and the PRODIGY study [18] (10% vs. 10.1%) found that 24-month DAPT had a similar risk of MACCE compared with 12-month DAPT. Sim et al. also found that extended DAPT failed to reduce the occurrence of MACCE (1.3% vs. 1.0%) in an observational study [9]. Furthermore, a recent report from the EXTEND-DAPT study used the data of the DAPT study combined with real-world data to reach a different conclusion from that of the DAPT study [8]. In EXTEND-DAPT, there was no longer a reduction in MACCE (reweighted treatment effect: –0.52, 95% CI –2.62 to 1.03) nor increased GUSTO bleeding (reweighted treatment effect: 1.15, 95% CI –0.08 to 2.45) with longer DAPT duration. The authors suggested the inconsistency between the real-world patients’ characteristics and those in the randomized clinical trials should be considered when making clinical decisions. Our real-world data support the above studies. We further provided evidence that DAPT should not be continued in Chinese ACS patients beyond one year after PCI.

Concurrently, we found an increased risk of revascularization in patients with prolonged DAPT (unadjusted HR: 2.97, 95% CI: 1.52–5.78), concordant with other studies conducted in China [10,19]. Since the regimen is chosen by the physician based on the patient’s condition, the patients with prolonged DAPT may have a higher risk of ischemia. However, the difference of ischemic risk factors has been minimized between the two groups. We consider that there may be other reasons for revascularization besides the higher risk of ischemia in >1 year group. Zheng et al. [20] found that prolonged DAPT causes intestinal damage, resulting in the induction of intestinal bacterial translocation into the bloodstream, increasing the incidence of ischemic events after PCI. This may be an explanation for the observed increase in revascularization events. This question needs to be further studied.

A large proportion of the patients included in the present study had unstable angina (69.89%), meaning that our patients may have had a lower risk of ischemia. Several studies in high-risk populations have found that extended DAPT may have certain benefits [19,21,22], which may relate to the different population distribution. Wang et al. pointed out that in patients with high thrombotic risk, long-term DAPT was associated with a reduced risk of MACCE (HR 0.38, 95% CI 0.27–0.54) and all-cause death (HR 0.07, 95% CI 0.03–0.15) [21]. The duration of DAPT for post-PCI patients may need to be tailored to each patient’s individual profile. As an observational study, the treatment regimens of the included patients were determined by physicians based on evaluation results. The guidelines have listed clinical and procedural factors associated with increased ischemia risk including advanced age, ACS presentation, Multiple prior MIs, Extensive CAD, Diabetes mellitus and CKD [1,2,3]. The above suggestions may be the major basis for physicians to choose treatment regimes. In present study, patients in the DAPT > 1-year group had a higher proportion of prior PCI, prior CABG, prior MI and diabetes than the DAPT = 1-year group with more stents and older age. It is consistent with the recommendations of the guidelines. To minimize the selection bias, we eliminated those baseline differences by PSM before statistical analysis. Nevertheless, we stratified the patients according to some cardiovascular risk factors in our study, but found no statistical differences in MACCE.

Several studies have developed scoring systems to guide whether to extend DAPT [23,24,25]. However, these scoring systems are mainly designed based on randomized clinical trials. Recent real-world applications of these scoring systems obtained varying results [26,27,28]. These paradoxical results suggest that guidance based on scoring systems may not be very accurate and should be considered with caution in clinical practice. Our study provides new evidence for the non-use of DAPT beyond 12 months.

Ticagrelor is a novel oral antiplatelet drug administered as a non-prodrug, and therefore avoids the effect of CYP2C19 loss-of-function genes. The PLATO study showed that ticagrelor combined with aspirin was associated with a lower risk of cardiovascular death, MI, and stroke than was clopidogrel combined with aspirin (9.8% vs. 11.7%) [29]. The presence of CYP2C19 loss-of-function alleles can render clopidogrel ineffective, which can lead to antiplatelet therapy failure or ischemic events. Furthermore, CYP2C19 loss-of-function alleles carriers are more general among East Asians than among Westerners [30]. Our previous study and numerous others have found that ticagrelor is superior to clopidogrel in carriers of CYP2C19 loss-of-function alleles [14,31]. The use of ticagrelor may significantly modify the effectiveness of antiplatelet therapy in Asians, but none of the previous studies examined extending a ticagrelor-based DAPT regimen. This study fills a void in previous studies, including patients who used ticagrelor (37.94%) for analysis. However, the results of extended DAPT did not vary according to the use of different P2Y12 inhibitors. We believe that switching to single-agent antiplatelet therapy 12 months after PCI should be considered regardless of which P2Y12 inhibitor is used.

This study also had some limitations: First, this study is a single-center study, and the obtained data may not be generalizable to all Chinese patients. Second, this is an observational study without randomization. The choice of DAPT duration is determined by the physician, which may lead to some bias. Although we used PSM to minimize baseline differences but some confounding factors may not have been considered. The study’s retrospective design might have led to potential bias. The included patients had tolerated 12 months of DAPT, which may mean the cohort consists of a large proportion of patients with low bleeding risk. This may have affected the outcomes of bleeding events. Third, due to the limitations of the database, the follow-up time of this study is insufficient to investigate the patient’s long-term survival status. Our study showed a trend toward lower risk of MACCE with extended DAPT but failed to show a significant benefit for patients within our study period. A more extended study period may change this situation.

## Conclusion

For ACS patients who have not experienced ischemia or bleeding within one year after PCI, prolonged DAPT does not appear to significantly reduce the risk of major cardiovascular and cerebrovascular ischemic events, nor does it increase the incidence of significant bleeding events within 18 months of follow-up. Prolonged DAPT may not bring sufficient benefits for ACS patients after PCI, but it may be related to an increased incidence of target vessel revascularization, which still requires further research.

## Data Accessibility Statement

The data that support the findings of this study are not available until the database is completed as the data also forms part of ongoing studies. In spite of this, the permission to access the data can be requested from the corresponding author by submitting a formal application. The data will be made available by the authors after the PHARM-ACS registry study is completed. A confidential and limited copy of data has been provided to the editor for review before publication.
